# Glyceraldehyde-3-phosphate dehydrogenase acts as an adhesin in *Erysipelothrix rhusiopathiae* adhesion to porcine endothelial cells and as a receptor in recruitment of host fibronectin and plasminogen

**DOI:** 10.1186/s13567-017-0421-x

**Published:** 2017-03-21

**Authors:** Weifeng Zhu, Qiang Zhang, Jingtao Li, Yanmin Wei, Chengzhi Cai, Liang Liu, Zhongmin Xu, Meilin Jin

**Affiliations:** 1 Animal Infectious Disease Unit, National State Key Laboratory of Agricultural Microbiology, Huazhong Agricultural University, Wuhan, China; 2 College of Animal Science and Veterinary Medicine, Huazhong Agricultural University, Wuhan, China; 3 Key Laboratory of Development of Veterinary Diagnostic Products, Ministry of Agriculture, Wuhan, China; 4 Cooperative Innovation Center for Sustainable Pig Production, Wuhan, China; 5 College of Life Sciences & Technology, Huazhong Agricultural University, Wuhan, China

## Abstract

*Erysipelothrix rhusiopathiae* is the causative agent of animal erysipelas and human erysipeloid. Previous studies suggested glyceraldehyde 3-phosphate dehydrogenase (GAPDH) plays a role in the pathogenesis of *E. rhusiopathiae* infection. We studied *E. rhusiopathiae* GAPDH interactions with pig vascular endothelial cells, fibronectin, and plasminogen. Recombinant GAPDH (rGAPDH) was successfully obtained, and it was shown that it plays a role in *E. rhusiopathiae* adhesion to pig vascular endothelial cells. Moreover, rGAPDH could bind fibronectin and plasminogen in a dose-dependent manner. To our knowledge, this is the first study demonstrating that a moonlighting protein plays a role in pathogenesis of *E. rhusiopathiae* infections.

**Electronic supplementary material:**

The online version of this article (doi:10.1186/s13567-017-0421-x) contains supplementary material, which is available to authorized users.

## Introduction, methods and results

*Erysipelothrix rhusiopathiae* is a small, Gram-positive, slender, straight, rod-shaped bacterium that can cause erysipelas in animals and erysipeloid in humans [1]. Swine erysipelas occurs worldwide and is of economic importance [1]. Acute swine erysipelas is characterized by septicemia and cutaneous lesions followed by sudden death. Histologically, vascular lesions can be observed in the systemic organs [1, 2]. Bacterial adhesion to endothelial cells may be a crucial event in the initiation of *E. rhusiopathiae* infection in swine [3].

Biological moonlighting refers to the ability of proteins to exert more than one function [4]. Many of these proteins appear to play roles in the virulence properties of bacteria or even in those of protozoan parasites and fungi [5–7]. The virulence roles of moonlighting proteins include all aspects of infection, such as attachment to host proteins, invasion, immune evasion, and immuno-modulation [4, 5, 8]. However, no study reports the roles of moonlighting proteins in pathogenesis of *E. rhusiopathiae* infections [9]. Thus, it is unknown whether moonlighting proteins exist in the *E. rhusiopathiae* infection process and what roles they play in pathogenesis. The first identified and best characterized moonlighting enzyme was glyceraldehyde 3-phosphate dehydrogenase (GAPDH) [4, 5]. A previous study demonstrated GAPDH was on cell surface of a *E. rhusiopathiae* strain [10]. If glycolytic enzymes are localized to the surface of microbial pathogens, they exhibit various functions unrelated to their housekeeping roles [5]. Thus, it is speculated that *E. rhusiopathiae* GAPDH play a role in the infection process. The aim of this study was to evaluate the ability of *E. rhusiopathiae* GAPDH to act as an adhesin in *E. rhusiopathiae* adhesion to pig vascular endothelial cells and as a receptor in *E. rhusiopathiae* recruitment of host fibronectin and plasminogen.

Glyceraldehyde 3-phosphate dehydrogenase gene (ERH_1534) [11] of *E. rhusiopathiae* epidemic virulent strain SE38 (isolated from heart of a pig that died from swine erysipelas in 2014) [12–14] was cloned and sequenced using primer GAPDH-seqF and GAPDH-seqL (Additional file 1). Sequence analysis indicated that the GAPDH gene (accession number KX714110) of the SE38 strain was identical to that of the *E. rhusiopathiae* Fujisawa strain [11]. Then *E. rhusiopathiae* GAPDH open reading frame (ORF) was cloned into pET-28a (+) using primers GAPDH-F and GAPDH-L (Additional file 1), expressed in *E. coli* BL21(DE3) (TransGene, Beijing, China) and purified by His-Trap chromatography according to the manufacturer’s protocols (GE Healthcare, Little Chalfont, Buckinghamshire, UK). SDS-PAGE analysis demonstrated that rGAPDH was successfully cloned, expressed and purified with an apparent molecular weight of approximately 39 kDa (Additional file 2). Then anti-rSpaA serum was successfully gained as previously described [15].

**Figure 1:**
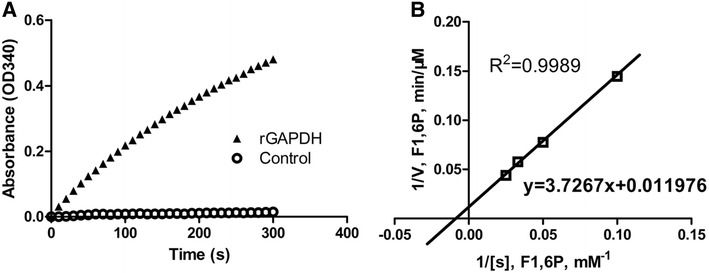
**Enzymatic characterization of purified rGAPDH. A** The rGAPDH enzymatic activity determined by measuring the conversion of NAD+ to NADH. **B** Michaelis–Menten kinetics (V_max_ and K_m_) of rGAPDH using a Lineweaver–Burk plot (double-reciprocal plot).

rGAPDH enzymatic activity was determined by measuring the transformation of NAD+ to NADH as previously described [16], with minor modifications. A reaction mixture (1 mL) containing K_2_HPO_4_ (100 mM; pH 7.4), fructose 1,6-bisphosphate (F1,6P; 40 mM), aldolase (10 U), EDTA (0.5 mM), NAD+ (10 mM), and purified rGAPDH (0.1 μg/mL) was measured by spectrophotometric assessment (A340) to determine NADH formation at 10-s intervals for 5 min. Negative controls were prepared as described above but without the addition of the rGAPDH. The specific activity of the recombinant protein was 228 μmol NADH/min/mg (Figure 1A), confirming the enzymatic functionality of the rGAPDH. The enzymatic kinetics of GAPDH were further determined using different F1,6P concentrations (10, 20, 30 and 40 mM). Then Michaelis constant (K_m_) and maximum reaction velocity (V_max_) of rGAPDH were determined according to double-reciprocal Lineweaver–Burk plots. The K_m_ for F1,6P was estimated to be 110 mM, and the V_max_ was 83.5 μM/min (Figure 3B), which were within the range reported for other microbial rGAPDHs [16–19].

**Figure 2:**
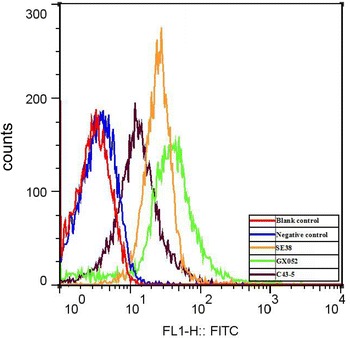
**Detection of** ***E. rhusiopathiae*** **GAPDH surface display using flow cytometry analysis.** Blank control, bacteria only treated with PBS; negative control, bacteria treated with preimmune serum; SE38, GX052, and C43-5: bacteria treated with anti-rGAPDH serum.

Flow cytometry analysis was used to detect GAPDH on the surface of three *E. rhusiopathiae* strains’ cells (two epidemic virulent strains: SE38 and GX052 and a classical virulent strain C43-5 [13, 14]) as previously described [20]. Briefly, overnight cultures [10^8^ colony-forming units (CFU)/mL] of different *E. rhusiopathiae* strains were incubated with mouse anti-rGAPDH serum (preimmune serum as control), and goat anti-mouse IgG–fluorescein isothiocyanate (FITC) (KPL) was used as secondary antibody. Then, the samples were detected using a flow cytometer (Becton–Dickinson, CA, USA). Significant mean fluorescence intensity (MFI) was detected in all three *E. rhusiopathiae* strains’ cells incubated with mouse anti-rGAPDH serum (Figure 2). The MFI of the *E. rhusiopathiae* treated with preimmune serum was distributed normally with a MFI close to that of unlabeled *E. rhusiopathiae*, whereas the MFIs of bacteria treated with anti-rGAPDH serum was approximately tenfold that treated with preimmune serum.

**Figure 3:**
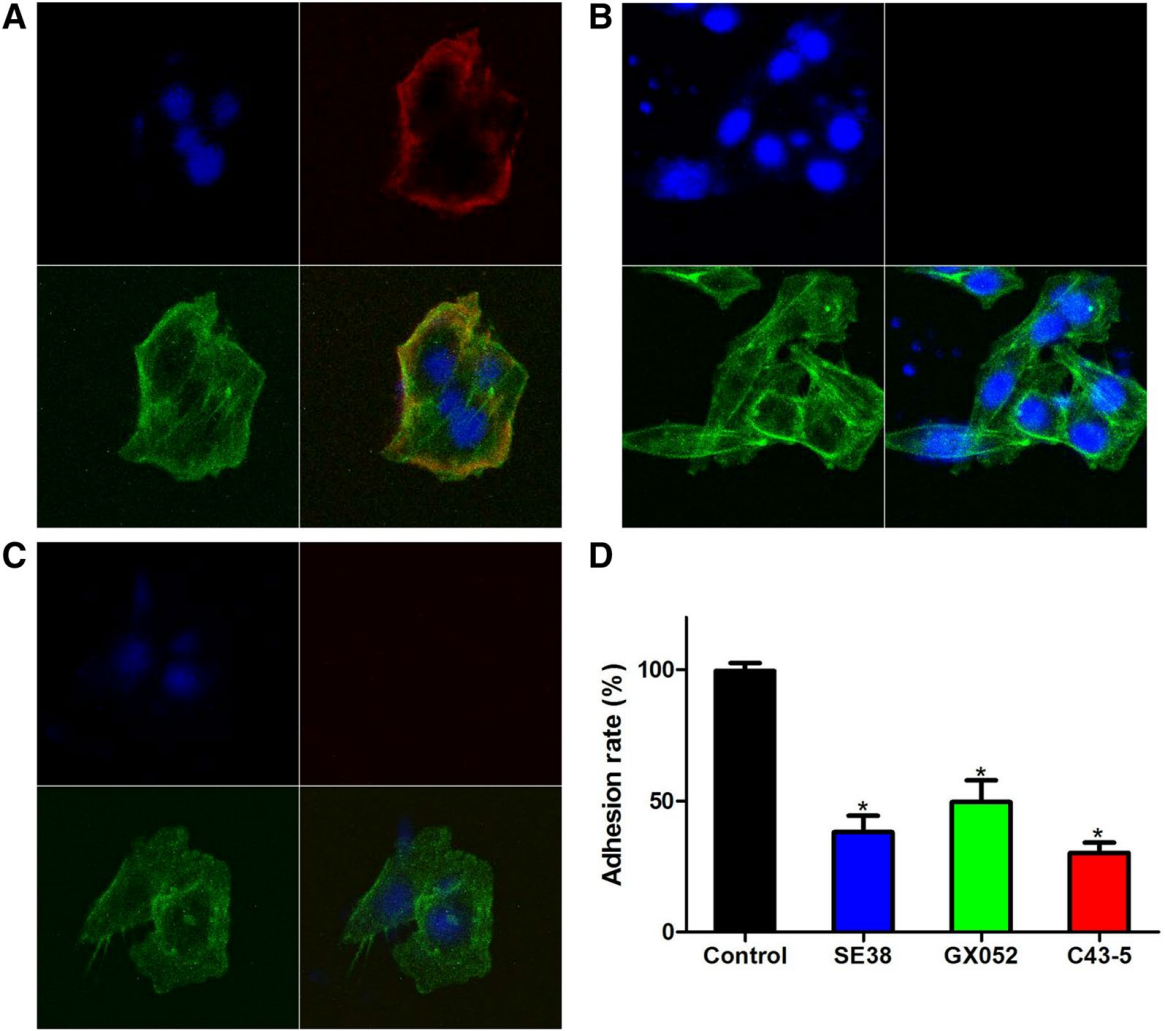
**Role of GAPDH in** ***E. rhusiopathiae*** **adhesion to PIECs.** **A**–**C** Blue color indicates the PIEC nucleus, green indicates PIEC microfilaments (actin), red indicates rGAPDH adhering to PIEC membranes. **D** Adhesion inhibition assay of *E. rhusiopathiae* to PIECs. Adhesion rate: number of CFU recovered in rGAPDH incubated group/number of CFU recovered in PBS incubated group × 100%. Data are expressed as mean ± SD of at least three experiments with samples in triplicate.

An indirect immunofluorescence assay was used to determine whether *E. rhusiopathiae* GAPDH can specifically adhere to the surface of Pig iliac arterial endothelial cells PIECs Cell (Cell Bank of the Chinese Academy of Sciences, Shanghai, China) [20]. Fixated PIECs were incubated with rGAPDH (10 μg). Mouse anti-rGAPDH serum (1/500) was used as primary antibody and goat anti-mouse IgG–Cy3 (Beyotime, Nanjing, Jiangsu, China; 1/500) was used as secondary antibody. At last, the microfilament (actin) were stained with phalloidan-FITC (Beyotime, Nanjing, Jiangsu, China) and cell nuclei were stained with 6-diamidino-2-phenylindole (DAPI). In negative control, rGAPDH was replaced by BSA or anti-rGAPDH serum was replaced by preimmune serum. Fluorescence was detected using a Zeiss LSM 510 laser scanning confocal microscope (zeiss, Mannheim, Germany). Red circles (Cy3) were found around (on cell membranes of) PIECs incubated with rGAPDH, whereas, no red circle was found in negative controls (Figures 3A–C). Thus it was thought rGAPDH could specifically bind to PIEC cell membranes.

A competitive adhesion inhibition assay was used to study the role of GAPDH in *E. rhusiopathiae* adhesion to PIECs [20, 21]. PIECs pre-incubated with rGAPDH were incubated (1:10) with the *E. rhusiopathiae* SE38, GX052 and C43-5 strains. After washes, the number (CFU) of *E. rhusiopathiae* adhering to PIECs was determined by viable counts. It was found that the adhesion rates of the three strains decreased significantly when *E. rhusiopathiae* adhere to PIECs pre-incubated with rGAPDH (Figure 3D).

**Figure 4:**
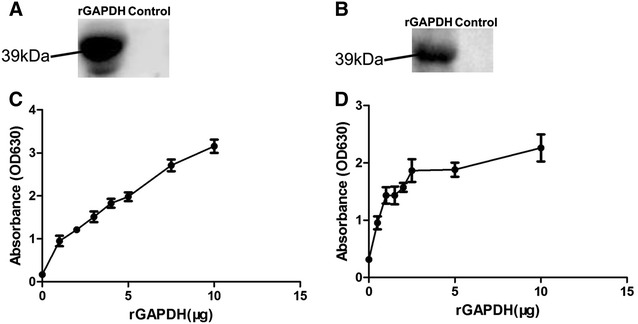
**Binding activity of rGAPDH to fibronectin and plasminogen. A**, **B** Far Western blot analysis of binding activity of rGAPDH to fibronectin and plasminogen; **C**, **D** ELISA analysis of binding activity of different concentrations of rGAPDH to immobilized fibronectin and plasminogen. Data are expressed as mean ± SD of at least three experiments with samples in triplicate.

*Erysipelothrix rhusiopathiae* GAPDH binding activity to fibronectin and plasminogen was detected using far Western blot assays [21, 22]. After SDS-PAGE, 10 μg of rGAPDH was transferred onto nitrocellulose (NC) membranes (BSA as a negative control). Then 10 μg/mL of human fibronectin (Sigma) or human plasminogen (Sigma) were incubated, with rabbit anti-fibronectin polyclonal antibody or rabbit anti-plasminogen polyclonal antibody (Boster, Wuhan, Hubei, China, 1:250) used as primary antibody, and goat anti-rabbit IgG–HRP (KPL, 1:5000) was used as a secondary antibody. At last, the membrane was developed with ECL Plus Western Blotting Detection System (Advansta, Menlo Park, CA, USA) and imaged on the Image Station 2000 MM (Kodak, USA). rGPADH could specifically bind fibronectin and plasminogen. Specific binding bands of 39 kDa were found in rGAPDH lanes, whereas no specific band was observed in the BSA lanes (Figures 4A and B).

*Erysipelothrix rhusiopathiae* GAPDH binding ability of fibronectin or plasminogen was also detected using enzyme-linked immunosorbent assays (ELISAs) [21, 22]. Briefly, 96-well microtiter plates were coated with 1 μg of fibronectin or plasminogen. Then, the wells were incubated with 50 μL of different concentrations of rGAPDH. Mouse anti-rGAPDH serum (1/500) was used as a primary antibody and HRP-conjugated anti-mouse IgG (KPL, 1/5000) was used as second antibody. The ELISA plate binding assay demonstrated that rGAPDH could bind fibronectin and plasminogen in a dose-dependent manner (Figures 4C and D).

## Discussion

Flow cytometry analysis was applied to determine the cell-surface display of GAPDH. The MFIs of three *E. rhusiopathiae* strains treated with anti-rGAPDH serum were significantly higher than that of bacteria treated with preimmune serum. The reason for this difference is the presence of the GAPDH antigen on the bacterial cell surface of the three strains examined here, which is well recognized by the mouse anti-rGAPDH antibody [20]. An immunogold electron microscopy analysis also demonstrated GAPDH is on surface of the *E. rhusiopathiae* Fujisawa strain [10].

We found that *E. rhusiopathiae* could adhere to PIECs via GAPDH. Adhesion to the host is the first step of infection, and adhesion to endothelial cells may be a crucial event for *E. rhusiopathiae* infection [3, 17]. Thus, our results suggested that GAPDH is an important candidate virulence factor of *E. rhusiopathiae*. However, GAPDH is not the only adhesin of *E. rhusiopathiae*. Other adhesins, such as SpaA, RspA and RspB [23–25], have been reported. Thus, in the competitive adhesion inhibition assay the inhibition was only partial. Our results demonstrated rGAPDH can also bind host fibronectin and plasminogen in a dose-dependent manner. Thus, GAPDH can act as a receptor in *E. rhusiopathiae* recruitment of fibronectin and plasminogen. This recruitment may play a role in colonization, invasion, inflammation and immune evasion processes, including adhesion to host cells, degradation of fibrin clots, influencing signaling pathways, and destroying immune effector molecules [26, 27].

In summary, the present study demonstrated that *E. rhusiopathiae* GAPDH acts as an adhesin in *E. rhusiopathiae* adhesion to PIEC and acts as a receptor in *E. rhusiopathiae* recruitment of host fibronectin and plasminogen. GAPDH is thus an important candidate virulence factor for *E. rhusiopathiae*. To our knowledge, this is the first report on involvement of moonlighting proteins in *E. rhusiopathiae* infection. The roles of moonlighting proteins in virulence include all aspects of infection [4, 5, 8]. Moreover, moonlighting proteins have been suggested to be broad-spectrum vaccine candidates [28, 29]. Thus, *E. rhusiopathiae* moonlighting proteins and their roles in infection and immunity should be further studied in the future.

## Additional files

**Additional file 1.** **Primers used in this study.** Primers used for sequencing and cloning *E. rhusiopathiae* GAPDH gene.

**Additional file 2.** **Analysis of rGAPDH expression and purification using SDS-PAGE followed by Coomassie blue staining.** Lanes: M, molecular size markers; Lane 1, crude extract from cells without expression vector; Lane 2, crude extract from uninduced cells carrying expression vector; Lane 3, crude extract from induced cells with 1 mM IPTG; Lane 4, (His)_6_ rGAPDH purified by Ni–NTA.
