# Supplementary material for: Glyceraldehyde-3-phosphate dehydrogenase acts as an adhesin in Erysipelothrix rhusiopathiae adhesion to porcine endothelial cells and as a receptor in recruitment of host fibronectin and plasminogen
Source: Vet Res. 2017 Mar 21;48:16. doi: 10.1186/s13567-017-0421-x (PMC5360030; doi:10.1186/s13567-017-0421-x)
Supplement: Supplementary file 1 — Additional file 1. Primers used in this study. Primers used for sequencing and cloning E. rhusiopathiae GAPDH gene. [file 13567_2017_421_MOESM1_ESM.doc]

**Additional file 1 Primers used in this study.**

| Primer | Sequence (5’-3’) | Amplicon size | Application |
| --- | --- | --- | --- |
| GAPDH-seqF | AATAGCCTTTATTTATGTACACT | 1160bp | Sequencing GAPDH gene |
| GAPDH-seqL | TAGTTGGCTCACAATTGACA |
| GAPDH-F | CGCGGATCCATGACAGTTAAAGTAGCAAT^a^ | 1023bp | Expressing GAPDH |
| GAPDH-L | CCGGAATTCTTAGAATTTTGAAGCAACGTAAT^b^ |

^a^ underlined sequence was introduced *Bam*HI site

^b^ underlined sequence was introduced *Eco*RI site
