# Supplementary figures and images for: Glyceraldehyde-3-phosphate dehydrogenase acts as an adhesin in Erysipelothrix rhusiopathiae adhesion to porcine endothelial cells and as a receptor in recruitment of host fibronectin and plasminogen
Source: Vet Res. 2017 Mar 21;48:16. doi: 10.1186/s13567-017-0421-x (PMC5360030; doi:10.1186/s13567-017-0421-x)

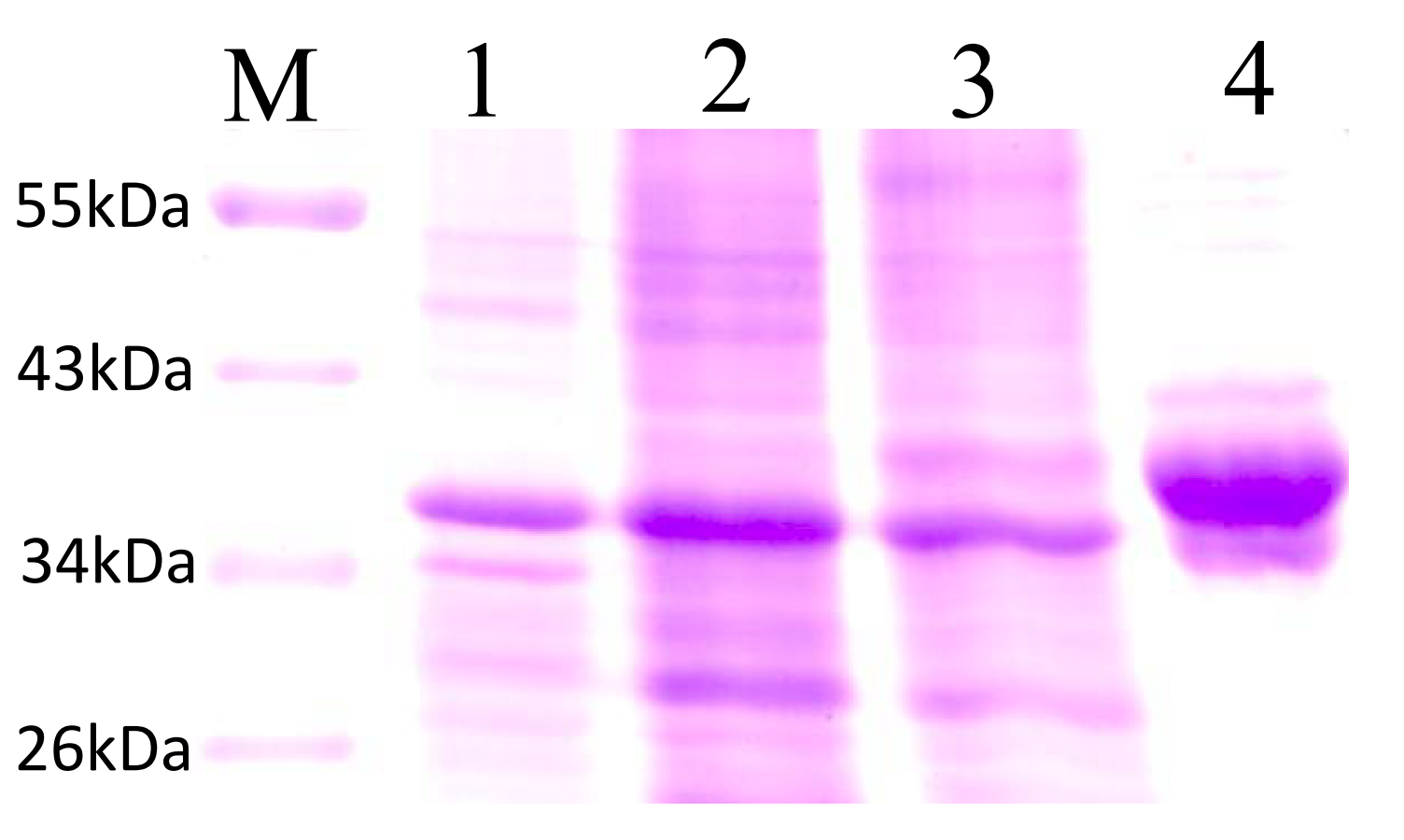

Supplement: Supplementary file 2 — Additional file 2. Analysis of rGAPDH expression and purification using SDS-PAGE followed by Coomassie blue staining. Lanes: M, molecular size markers; Lane 1, crude extract from cells without expression vector; Lane 2, crude extract from uninduced cells carrying expression vector; Lane 3, crude extract from induced cells with 1 mM IPTG; Lane 4, (His)6 rGAPDH purified by Ni–NTA. [file 13567_2017_421_MOESM2_ESM.tif]
